# Perforated Intestine—Artificial Anus Cured

**Published:** 1826-07

**Authors:** 


20. *Perforated Intestine—Artificial Anus cured.* A farmer, aged 40 years, of strong constitution, complained of most violent pains in the abdomen, accompanied by vomiting. His groins were examined, and in the left, was found a small tumour, which had made its appearance there suddenly while carrying a heavy burthen some time previously. This swelling had given no inconvenience till the present time. The physician, not being able to reduce the swelling, contented himself with ordering some enemata, diluent drink, and a cataplasm to the part. *Second day.* Same symptoms. *Third day.* The symptoms were exasperated. There were hiccup—vomiting—extreme tenderness of the tumour—distention of the abdomen—feeble pulse. Not doubting that the case was a strangulated hernia, a surgeon was sent for. The fourth and fifth days were passed in a sad state, as no surgeon could be found to operate. On the *sixth* day, the physician was suddenly called, and found the swelling just bursting, and discharging a quantity of thick white pus. *Seventh day.* The patient is doing well—much suppuration—in the interior of the abscess could be seen a portion of epiploon half mortified. This was cut away, and a portion of intestine was found adherent to the edge of the wound. The bad symptoms again returned with full force—and on the 9th day a living lumbricus, six inches in length, presented itself at the wound, and was drawn out by the forceps. *10th and 11th days.* Nothing particular occurred. *12th day.* The patient complained of violent colicky pains, and on removing the dressings, excrementitious matters issued forth, together with another lumbricus of the same size as the first. *13th.* Abundant stools were this day procured by some castor oil, and a great amelioration of the symptoms succeeded. Fæces continued to be discharged from the wound, which had now considerably increased in size. From the *13th* till the *16th day*, the patient experienced no other inconvenience than the constant passage of fæcal matters by the wound. Our author then had recourse to the plan of Desault, and placed a pad of charpee on the part, supported by an elastic bandage. No inconvenience followed, and two days afterwards, motions were passed per anum. From this time granulations went on forming in the wound, till it was entirely cicatrised, and the man was able to return to his usual avocations.

The question is, had the worms any part in the breach of structure in the intestine? We think it improbable, though not impossible. The delay of the operation, in this case, as in one recorded in our last Number, is not creditable to French surgery, in the provinces; because it is well known that the French physician receives the same professional education as the surgeon, and *vice versa.* The same should be the case, indeed, every where. —*Revue Med. Jan.* 1826.

